# Bioactive Compounds of *Opuntia* spp. Acid Fruits: Micro and Nano-Emulsified Extracts and Applications in Nutraceutical Foods

**DOI:** 10.3390/molecules26216429

**Published:** 2021-10-25

**Authors:** Fabián Fernández-Luqueño, Gabriela Medina-Pérez, Elizabeth Pérez-Soto, Salvador Espino-Manzano, Laura Peralta-Adauto, Sergio Pérez-Ríos, Rafael Campos-Montiel

**Affiliations:** 1 Sustainability of Natural Resources and Energy Programs, Cinvestav-Saltillo, Coahuila C.P. 25900, Mexico; cinves.cp.cha.luqueno@gmail.com; 2 Institute of Agricultural Sciences, Autonomous University of the State of Hidalgo, Hidalgo C.P. 43600, Mexico; gabriela_medina@uaeh.edu.mx (G.M.-P.); epsoto@uaeh.edu.mx (E.P.-S.); pe262989@uaeh.edu.mx (L.P.-A.); sergiorpr@gmail.com (S.P.-R.); 3 Food Agroindustrial Area, Xicotepec University of Juarez Technology University, Avenida Universidad Tecnológica #1000, Tierra Negra, Xicotepec de Juárez, Puebla C.P. 73080, Mexico; salvador.espino@utxicotepec.edu.mx

**Keywords:** antioxidant, antibacterial, hepatoprotective, gastroprotective, nanoemulsions, encapsulation, in vitro, functional food

## Abstract

The acid fruit of the "xoconostle" cactus belongs to the genus *Opuntia* family of cacti. It is used as a functional food for its bioactive compounds. Several studies reported that xoconostle fruits have a high amount of ascorbic acid, betalains, phenols, tannins, and flavonoids. These compounds confer antioxidant, antibacterial, anti-inflammatory, and hepatoprotective gastroprotective activity. Xoconostle fruit extracts were tested by in vitro assays where the digestion conditions were simulated to measure their stability. At the same time, the extracts were protected by encapsulation (microencapsulation, multiple emulsions, and nanoemulsions). Applications of encapsulated extracts were probed in various food matrices (edible films, meat products, dairy, and fruit coatings). The xoconostle is a natural source of nutraceutical compounds, and the use of this fruit in the new food could help improve consumers’ health.

## 1. Introduction

The fruit of xoconostle (acid prickly pear) can be light green, pink, or red. It contains sugars, vitamin C, phenolic compounds, carotenoids, and betacyanins, all functional ingredients. The fruit’s mesocarp has been reported to contain a 30–40% fiber content, and the seeds have a high fiber content [1]. Xoconostle possesses bioactive compounds such as betacyanins and phenolic compounds [2]. It is preserved for several months in the plant without deterioration, and even for several weeks in cool and dry places, without losing its flavor, color, and humidity properties; this is due to its low pH (3.7–4.5), which also supports its commercialization [3].

This fruit belongs to the Cactaceae family of the *Opuntia* genus, the most diverse genus in America with a value of between 191 and 215 species; it is worth mentioning that both the genus and the Cactaceae family are endemic to the American continent [4]. The production of xoconostle is distributed in areas with diverse edaphic-climatic conditions [5]. The most important extensions of xoconostle-producing cactus plants are grown in a warm climate (16–22 °C) that is moderately arid (300–600 mm of water per year) [6]. Its maturation time varies between species; Martínez-González et al. [7] mention that *O. joconostle* and *O. leiascheinvariana* mature in November of the same year, *O. mutudae* in March of the following year of flowering, and *O. oligacantha* in October of the same year of flowering.

*Opuntia* species have a high level of hybridization and subsequent speciation via polyploidy [8]. In Mexico, ten species have been described to produce xoconostle fruits: nine belong to the genus *Opuntia* (*Opuntia heliabravoana* Scheinvar, *Opuntia elizondoana* E. Sánchez and Villaseñor, *Opuntia joconostle* FAC Weber, *Opuntia matudae* Scheinvar, *Opuntia spinulifera* Salm-Dyck, *Opuntia leucotricha* DC, *Opuntia zamudioi* Scheinvar, *Opuntia durangensis* Britton and Rose, *Opuntia oligacantha* C.F. Förster) O. *joconostle* F.A.C. Weber ex Diguet cv.

Cuaresmeño is the most exploited and commercialized, followed by O. *matudae* Scheinvar cv. Rosa. The beneficial effects of this fruit are due to its antioxidant activity related to the composition and concentration of phenolic compounds. The "Cuaresmeño" xoconostle (O. *matudae*) is the most commercialized and consumed type around the world; it is one of the best characterized, reporting soluble phenols, ascorbic acid, betalains, and carotenoids as functional constituents [1]. Mexico produces approximately 10,000 tons per year [9].

Xoconostles are fruits whose outer wall is thin, while their inner walls are thick, edible (with an acidic flavor), and represent three-quarters of the fruit. Unlike prickly pears, the seeds of the xoconostle are in the center of the fruit—on the other hand, in the prickly pear, they occupy almost the entire width of the structure [1].

**Figure 1:**
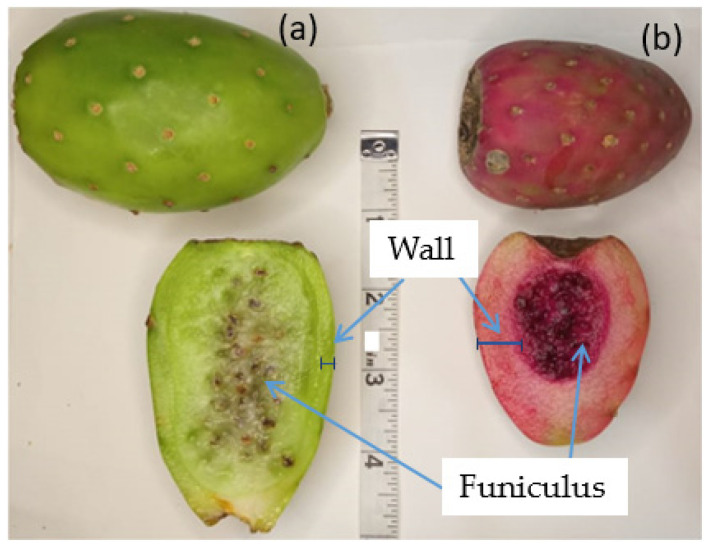
Distinctive characteristics between prickly pear cactus fruit (**a**) and xoconostle (**b**).

The acid prickly pear is a cylindrical, pear-shaped, or spherical berry with an approximate weight of 60 g, a diameter of approximately 3.6–5 cm, and an apical receptacle. Morphologically, the xoconostle structure can be divided into three layers: pericarp, also called peel, representing 20–24% (of the total structure); mesocarp or pulp, which is the edible part (58–64%); and endocarp, where the seeds are embedded in a mucilaginous matrix (13–18%) (Figure 1) [10].

Several studies reported that the consumption of xoconostle improves health by preventing the development of chronic diseases, such as diabetes, and other health problems, such as obesity and respiratory diseases, due to the high content of antioxidants [1]. Xoconostle and its by-products (seeds and shell) could be used as a functional ingredient for the food industry, enriching bioactive compounds such as tocopherols, ascorbic acid, and phenolic compounds [11]. Due to its composition and distinct morphology, various studies focus on endorsing the importance of xoconostle due to its nutritional and functional capacity. It has been analyzed by fractions (peel, pulp, seed, and whole fruit). As a result, the fruit’s healthful properties have been identified: hypoglycemic, lipid-lowering, hypocholesterolemic, anti-inflammatory, antiulcerogenic, immunostimulating, antimicrobial, antioxidant, and antioxidant, antidiabetic activity [1]. Xoconostle has contributed to traditional Mexican medicine since its pulp and peels have been used because it was attributed a hypoglycemic effect and an adjunct in controlling cholesterol and reducing obesity [8]. It is also used as a cough remedy to reduce symptoms of the flu, to treat diabetes and blood pressure (the peel and nopal of the fruit are consumed in a liquefied beverage), as a laxative (only the peel), and to heal bruises and tumors (whole fruit) [2,12,13].

## 2. Chemical Composition of Xoconostle Fruit 

The fruits of xoconostle (as observed in Table 1) commonly possess minerals (calcium, iron, magnesium, potassium, zinc, and phosphates); vitamins (A, B1, B2, and C); and polyphenols, carotenoids, and betalains [1,2,14,15,16]. Currently, research is being carried out on the nutritional and functional content of different varieties of xoconostle. On the other hand, Núñez-Gastélum et al. [17] determined that the seeds of *O. polyacantha*, *O. engelmannii*, *O. phaeacantha*, and *O. macrocentra* can have an approximate water content of 4.23–5.88%, minerals 2.5–3.2%, protein 10.45–14.83%, lipids 9.23–10.45%, carbohydrates 67–72%, and a total of phenolic compounds in the range of 10.78–13.2%.

### Bioactive Compounds

Bioactive compounds are essential substances since they play an essential role in our homeostatic balance. Some of these include vitamins (A, C, and E), phenols, flavonoids, carotenoids, betalains, alkaloids, and tannins; they are present in plant foods such as fruits, vegetables, vegetables, cereals, and some spices [19]. Thus, it is essential to eat a diet accompanied by these nutritional and functional foods.

Phenols are chemically defined as substances with an aromatic ring with one or more hydroxyl groups (OH), including their functional groups; these can be monophenols and polyphenols depending on their structure [20]. These compounds are present in plants because they are essential for plant development and the protective mechanism, for example, against UV light and damage by phytopathogenic organisms [21]. Furthermore, their inclusion in the structure of vegetables can be used for our physiological benefit. Bioactive compounds are species that act according to the substrate they face, eliminating free radicals thanks to their antioxidant capacity; they can also chelate metals and inhibit the activity of some indicator enzymes of various physiological damages [22].

Currently, there are studies on the nutritional and functional content of different varieties of xoconostle. In addition, some have focused on analyzing the chemical composition of their structure separately (endocarp, mesocarp, and whole fruit) (Table 1).

The xoconostle is a fruit characterized as pale green, pink, or red depending on the species; it has a great nutritional value, represented by containing sugars, phenolic compounds, carotenoids, and Betacyanins [10].

Regarding the pH of xoconostle, it is less than 3.5; the low pH prevents the growth of harmful microorganisms, which constitutes an advantage concerning the safety of the products, which supports its use as a condiment in gastronomy [23]. Yahia and Mondragón-Jacobo [24] differentiated the acidic prickly pear (O. spp) from the sweet prickly pear, by its low content of soluble solids of 4.0°–5.9° Brix when compared to the prickly pear (*Opuntia ficus-indica*), which had a value of 11.6°–15.3° Brix. [2].

Hernandez Fuentes et al. [2] studied the chemical composition and antioxidant and mineral profile of 10 different varieties of xoconostle, finding significant differences in all characterization analyzes. Monroy-Gutiérrez et al. [25] evaluated the total phenol content, the ascorbic acid content of xocotuna, xoconostle, and prickly pear species. Regarding the content of pigments such as betalains, total chlorophyll, and carotenes, high variability was observed due to the characteristic color of the different cultivars. The higher levels of dietary fiber content have been reported in the pulp of Cuaresmeño xoconostle (*Opuntia matudae*) from 30 to 34% [1,26].

Moreover, edible flowers are rich in bioactive compounds such as anthocyanins, vitamins, carotenoids, polyphenols, and flavonoids [27]. Flowers of xoconostle have been studied; Pensamiento-Niño et al. [28] characterized the nutritional and chemical composition of xoconostle flowers (cardon xoconostle (*Cylindropuntia rosea*), Ulapa xoconostle (*Opuntia oligacantha*), and pink Lenten xoconostle (*Opuntia matudae*). They identified the presence of quercetin and isorhamnetin and derivatives such as quercetin 3-*O*-acetyl-rhamnoside, protocatechuic acid 4-*O*-glucoside, isorhamnetin 3-*O*-glucoside, isorhamnetin 3-*O*-7-*O*-rhamnoside glucoside, apigenin 6,8-di-*C*-glucoside, and quercetin 3-*O*-xylosyl-glucuronide in xoconostle flowers. The differences in the nutritional and chemical composition of xoconostle can be attributed to the characteristics of the different genotypes of both species and cultivars and geographical conditions [29,30]; some of the published results from characterized fruits are summarized in Table 2. 

## 3. Antioxidant and Antibacterial Activity of Xoconostle Extracts

Santos-Díaz et al. [32] reported that vegetative structures of *Opuntia* spp. (fruit, roots, cladodes, seeds, and juice) could present functional properties due to the high content of bioactive compounds. They have been investigated in different cell and animal models and in human clinical trials, which makes it possible to characterize and clarify the protective effect of opuntia-enriched diets against pathologies.

Hernández-Fuentes et al. [2] determined that "xoconostle" acid fruits are notably different compared to other cactus fruits. Xoconostle fruits contain bioactive compounds such as ascorbic acid, tocopherols, phenolic compounds, flavonoids, and pigments (carotenoids and betacyanins). These essential compounds perform three main functions: antioxidant potential, metal chelation, and inhibition of disease-triggering enzymes. The antioxidant capacity prevents oxidation reactions, thus avoiding the oxidative damage that occurs in the exposure of cells to various sources, causing a breakdown of the balance between pro-oxidant substances and the antioxidant mechanisms responsible for eliminating said chemical species [33].

Xoconostle (*Opuntia oligacantha* C.F. Först) has also been shown to have antimicrobial, antifungal, and antioxidant effects thanks to its content of phenolic acids and flavonoids. Authors have reported antimicrobial, antifungal, and antioxidant activity; xoconostle could be an excellent alternative in various processes to prove food conservation and nutritional quality [34].

Similarly, Espinosa Muñoz et al. [35] evaluated the antimicrobial activity of xoconostle (*Opuntia oligacantha*) through ultrasound-assisted extraction [35]. The extracts of xoconostle had inhibitory activity against *Salmonella typhimurium* and *Staphylococcus aureus.* Thus, this type of study verifies the use of xoconostle extracts as a natural antibacterial additive in the food industry.

## 4. Potential Technological Applications of Xoconostle

A growing interest in the development of active food packaging exists that, in addition to fulfilling its packaging functionality, also provides advantages in the preservation of shelf life and the nutritional and functional properties of the food [36]. Examples of these packages are oxygen scavengers, carbon dioxide emitters or absorbers, ethanol emitters, moisture absorbers, flavor-release systems, light-absorption or -regulation systems, time-temperature indicators, and anti-fog and anti-adherence films [37]. The new trend of the food industry during the last years seeks to replace the production of food packaging based on synthetic materials by developing innovative packaging based on polymers of natural origin, such as polysaccharides and proteins [33]. This is achieved with some types of active packaging that produces a controlled release of bioactive compounds (with antimicrobial and antioxidant bioactivity), avoiding their direct application of active agents to food [38]. The addition of these bioactive compounds to biodegradable films helps preserve the functional properties of the food product. In addition, it provides safety and quality, such as the use of microencapsulation and nanoencapsulation [36].

Microencapsulation is a technology for packaging solid, liquid, or gaseous materials that extend the product’s useful life. In addition, this process is appropriate for heat-sensitive materials [38]. An example of this is the application of starch films enriched with natural antioxidants to maintain food quality through the same potential; this application turns out to be an innovative solution in the food industry [39]. Cenobio-Galindo et al. [40] developed starch films where they microencapsulated *O. oligacantha* extract and studied the antioxidant and antibacterial activity against *Salmonella typhimurium*. It is worth mentioning that the films with the xoconostle extract were more efficient regarding mechanical properties (thickness, tensile strength, percentage of elongation, and color). The microencapsulated films presented higher functional properties than the films with the extract. In a study on microencapsulation by spray-drying with microcapsules of *Opuntia* spp. (xoconostle and prickly pear) fruit extract obtained at 175 °C using mesquite gum as a wall material, the effect of spray-drying protected the content of total phenols, flavonoids, and pigments such as betalains; it is worth mentioning that xoconostle extracts behaved more resistant to degradation by spray-drying [33].

Nanocomposites act as carriers of bioactive compounds; the nanoparticles in the polymer matrix can bind to diverse molecules, thus increasing the container’s efficiency [41].

Espino-Manzano et al. [16] studied the application of xoconostle-extract/orange oil (*w*/*o*) nanoemulsions into gelatin films. They found a higher presence of phenolic compounds (41.31 ± 3.71 mg GAE/100 g), flavonoids (28.03 ± 3.25 mg EQ/100 g), and betalains 0.014 mg/g due to the film. Higher radical inhibition was also observed, 72.13% for 2,20-azino-bis-3-ethylbenzothiazoline-6-sulfonic acid (ABTS) and 82.23% for 1,1-diphenyl-2-picrylhydrazyl (DPPH). Both mixed in the gelatin film, bioactive compounds from the xoconostle extract, and orange oil act as a barrier against oxidation and microbiological and physical damage.

Nowadays, people are more attentive and interested in consuming functional foods to preserve and/or improve their health. For this reason, innovating foods containing bioactive compounds of natural origin, which provide benefits such as antioxidant activity and other bioactivities that improve specific diets, are highly recommended. Another development is the interest in the application of edible films to prolong the shelf life of the food and improve its sensory characteristics while maintaining the quality of the product. For example, Medina-Pérez et al. [42] incorporated nano-encapsulated compounds from xoconostle extract (*Opuntia oligacantha* C.F. Först) into chayotextle starch (*Sechium edule* Sw.) films; as a result, they obtained a significant content of phenols, but there was a decrease in the content of flavonoids. Furthermore, inhibition was observed against gram-negative bacteria (*Escherichia coli* and *Salmonella typhimurium*).

Campos-Montiel et al. [43] analyzed and evaluated the application of a double-emulsion system added to the formulation of pork meat products; the double emulsion was made from xoconostle extract, canola oil, and whey protein. This alternative significantly improved the physicochemical, nutritional properties (higher moisture and protein content and reduced saturated fat content), antioxidants, and the texture of emulsified meat products. These findings were also applied to dairy foods. Perez-Soto et al. [44] incorporated micro and nanoemulsions of xoconostle extract (*Opuntia oligacantha* C.F. Först) into fresh cheese curd. They maintained the final product for 45 days at 4 °C to evaluate the shelf life. The results showed that the addition of micro-and nanoemulsions did not affect the physicochemical parameters of the cheese curd.

Regarding microbiological inhibition studies, the antimicrobial effect was higher when nanoemulsions were added. At the same time, the addition of microemulsions positively influenced antioxidant activity. The addition of these systems changed the texture profile, with micro and nanoemulsion influencing hardness (Control: 8.60 ± 1.12, Micro: 1.61 ± 0.31, and Nano: 3.27 ± 0.37 N). 

Another work with dairy food was the development of a yogurt fortified with natural pigments (betalains) and antioxidant compounds (polyphenols) from xoconostle extract encapsulated in a multiple emulsion (EM) (*W1*/*O*/*W2*), which was analyzed for 36 days of useful life. The use of EM did not affect the viability of BAL, and, in addition, it protected the functional compounds [40,45]. Other researchers applied high-pulsation electric fields (HPEF) to the mesocarp and endocarp of nine varieties of prickly pears (*Opuntia* spp.). They reported that applying this method improves the profile of the bioactive compounds during a juice-extraction performance [46].

Nanoemulsions with xoconostle extracts were applied as an avocado coating. Nanoemulsions of 25 and 50% increased the shelf life by reducing weight loss and maintaining the firmness of the avocado; in addition, they contributed to reducing the activity of polyphenol oxidase, consequently reducing the browning of the avocado. Compounds such as phenols and flavonoids and antioxidant activity were maintained for 60 days. The histological study showed that the nanoemulsion delayed the maturity of the epicarp [47]. Xoconostle was used as an additive in vitro digestibility with ruminal liquid to reduce greenhouse gases. Different concentrations of xoconostle were used (0, 1.5, 3, and 4.5%). Xoconostle contains phenolic compounds that inhibit microorganisms. The addition of 4.5% of pulp decreased methane production in the in vitro trial (fermentation with corn stubble) [15].

## 5. Potential Nutraceutical Health Effects of Xoconostle Consumption

Nutraceutical properties of *Opuntia* spp. are widely documented. However, the acidic fruits (xoconostle) are little studied regarding clinical profiles in response to their consumption. Pimienta-Barrios et al. [48], conducted a clinical trial in which the effect of xoconostle skin intake was measured in human patients in two groups: healthy and those who have type two diabetes (DM2). This study took blood samples at different intervals after ingesting xoconostle, finding that peel consumption caused a statistically significant decrease of cholesterol and triglycerides in healthy people, increasing both glucose and insulin levels compared to no consumption. They reported that in the participants with DM2, xoconostle consumption diminished glucose concentration and increased insulin. These findings have also been studied by Medina-Perez et al. [14] who used in vitro α-amylase and α-glucosidase simulated digestibility assays. Inhibitory effects of extracts of *Opuntia oligacantha* C.F. Först peel, pulp, seeds, and whole fruit were measured; the whole-fruit extracts presented the highest inhibition activity (25 mg/mL with an inhibition of 63.0 ± 0.53%). This inhibitory effect could be because flavonoids can bind to biological polymeric enzymes such as α-amylase and α-glucosidase, inhibiting their enzymatic activity [49,50]. The above means that xoconostle could be used as a therapeutic strategy in controlled diets for diabetic patients and, of course, for the general public.

Paiz et al. [51] conducted a study in diabetic rats; it was found that total cholesterol and HDL levels were statistically similar among diabetic and healthy animals. A reduction (*p* < 0.001) in glucose concentration was observed in both healthy and diabetic rats dosed with the three *O. joconostle* supplements; this effect was most evident with the mesocarp (72%). Triglycerides only decreased (*p* < 0.001) in healthy rats. The effect of xoconostle consumption in hypercholesterolemic mice was studied by Osorio Esquivel et al. [52]. When the authors added methanolic extract obtained from *Opuntia joconostle* seeds to mice’s diet (1, 2, and 5 g/kg), the weight of mice was modified (*p* ≤ 0.001), and total cholesterol, low-density lipoprotein, triglycerides level, and atherogenic index were reduced. Although similar concentrations of HDL cholesterol were found in the control group, the authors explain that this activity could be the result of the seeds’ phenolic composition. The researcher did not report the toxic effects of the consumption of xoconostle and by-products. 

Medina-Perez et al. [53] evaluated gastroprotective, anti-inflammatory, and hepatoprotective activities of different parts of xoconostle fruit (*Opuntia Oligacantha* C.F. Först) by establishing in vitro simulated gastrointestinal conditions. Different extracts, pericarp, mesocarp, endocarp and whole fruit, urease, elastase, and β-glucuronidase, were tested and obtained the highest inhibition activity (86%, 79%, and 84%), respectively. Furthermore, bioactive compounds after in vitro gastrointestinal tests were maintained above 60% enzymatic inhibition activity.

## 6. Conclusions

Xoconostle content of bioactive compounds such as ascorbic acid, betalains, phenols, and flavonoids provides to other foods several properties: antioxidant, antibacterial, antidiabetic, anti-inflammatory, hepatoprotective, and gastroprotective activity. Moreover, these bioactive compounds can be included in different matrices, such as edible films of animal and vegetable origin and meat and dairy products, to produce bioactive films and functional foods. The development of micro-and nano-encapsulations has been a trend in the industry in recent years, so applying this technology in the preservation of bioactive compounds will help functionalize foods that are not considered healthy but are highly consumed. On the other hand, future research may measure the effects of xoconostle consumption on human or other species’ microbiota and synergic effects with compounds such as dietarian fiber.

## Figures and Tables

**Table 1:** Chemical composition of two varieties of xoconostle (*Opuntia* spp.).

	CV. Cuaresmeño		CV. Rosa		Reference
	Pulp	Seeds	Pulp	Seeds	[1]
	g/100 g		g/100 g		
Humidity	93.24 ± 0.02	73.95 ± 1.09	94.11 ± 0.00	60.44 ± 0.66	
Proteins	0.66 ± 0.01	2.12 ± 0.00	0.56 ± 0.00	3.45 ± 0.02	
Lipids	0.03 ± 0.0	2.45 ± 0.05	0.04 ± 0.00	3.52 ± 0.12	
Carbohydrates ^B^	3.69	1.71	3.93	1.56	
Soluble sugars	2.02 ± 0.09	0.95 ± 0.09	1.56 ± 0.17	1.47 ± 0.19	
Fructose	1.38 ± 0.03	0.71 ± 0.07	0.87 ± 0.03	0.99 ± 0.12	
Glucose	0.37 ± 0.05	0.15 ± 0.01	0.35 ± 0.13	0.34 ± 0.05	
Sucrose	0.27 ± 0.01	0.09 ± 0.00	0.34 ± 0.01	0.14 ± 0.02	
Total dietary fiber	2.31 ± 0.12	19.22 ± 0.15	1.74 ± 0.07	30.1 ± 0.64	
Insoluble fiber	1.45 ± 0.07	18.85 ± 0.12	1.16 ± 0.01	29.04 ± 0.57	
Soluble fiber	0.86 ± 0.05	0.36 ± 0.03	0.58 ± 0.07	1.13 ± 0.07	
	Whole fruit				[2]
Mineral content	mg 100 g^−1^ FW		mg 100 g^−1^ FW		
Ca	0.143 ± 0.07		N/D		
Mg	0.081 ± 0.003		N/D		
Fe	0.060 ± 0.006		N/D		
K	0.126 ± 0.0027		N/D		
Zn	0.0030 ± 0.0001		N/D		
Acids	mg 100 g^−1^ FW		mg 100 g^−1^ FW		[18]
Malic	179.2 ± 0.51		276 ± 0.5		
Citric	2650 ± 2.33		1309 ± 0.8		
Fumaric	17.67 ± 0.06		13.92 ± 0.07		
Oxalic	79.54 ± 1.84		46.23 ± 0.66		
Ascorbic	54.24 ± 0.86		16.35 ± 0.26		

^B^ Total carbohydrates were calculated as the difference of the summary of moisture, protein, fat, ash, and fiber values.

**Table 2:** Bioactive compounds in *Opuntia* spp. acid fruits.

Fruit Color	Cultivar	Structure	Total Phenols	Betalains		Antioxidant Activity			Total Flavonoids	Reference
				Betacyanins	Betaxanthins	ABTS	DPPH	Trolox		
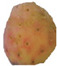	*Opuntia joconostle* F.A.C. Weber ex Diguet. (cv. Cuaresmeño)	Pulp (mesocarp)	38.57 ± 6.87 mg/100 g (FWB)	ND	ND	ND	5.14 ± 0.20 mg/mL of extract	ND	3.93 ± 0.19 mg CE/g of extract	[1]
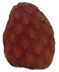	*Opuntia matudae Scheinvar* (cv. Rosa)	Pulp (mesocarp)	33.71 ± 2.09 mg/100 g (FWB)	ND	ND	ND	>16 mg/mL of extract	ND	0.86 ± 0.09 mg CE/g of extract	[1]
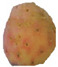	*Opuntia joconostle* F.A.C. Weber ex Diguet. (cv. Cuaresmeño)	Seeds [10] (Endocarp)	50.43 ± 4.86 mg/100 g (FWB)	ND	ND	ND	1.53 ± 0.05 mg/mL of extract	ND	24.18 ± 1.69 CE/g of extract	[1]
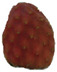	*Opuntia matudae* Scheinvar (cv. Rosa)	Seeds (Endocarp)	59.48 ± 0.69 mg/100 g (FWB)	ND	ND	ND	1.88 ± 0.11 mg/mL of extract	ND	58.40 ± 0.78 mg CE/g of extract	[1]
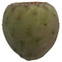	*Opuntia matudae*	Peel (pericarp)	863 ± 67 mg GAE/100 g (DWB)	0.59 ± 0.01 mg/100 g (DWB)	4.10 ± 0.28 mg/100 g (DWB)	ND	ND	14.5 mmol of Trolox equivalents/100 g (FWB)	ND	[9]
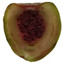	*Opuntia matudae*	Pulp and seeds (mesocarp and endocarp )	128 ± 6 mg GAE/100 g (DWB)	0.49 ± 0.00 mg 100 g^−1^ (DWB)	2.23 ± 0.11 mg 100 g^−1^ (DWB)	ND	ND	6.87 mmol of Trolox equivalents/100 g (FWB)	ND	[9]
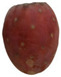	*Opuntia joconostle*	Whole fruit	13.08 ± 0.65 mg GAE/g (DWB)	27.98 ± 0.64 mg 100 g^−1^ (DWB)	ND	32.79 ± 1.42 mmol TE/100 g (DWB)	4.94 ± 0.64 mmol TE/100 g (DWB)	ND	1.19 ± 0.03 mg CE/g (DWB)	[31]
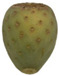	*Opuntia matudae* Scheinvar cv. Blanco”	Whole fruit	29.61 mg GAE/g (DWB)	ND		0.95 mg VCEAC g^−1^	ND	ND	ND	[25]
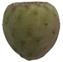	*Opuntia matudae* Scheinvar cv“Cuaresmeño”	Whole fruit	44.61 mg GAE/g	1.18 mg 100 g^−1^	0.34 mg 100 g^−1^	0.96 mg VCEAC g^−1^	ND	ND	ND	[25]
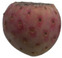	*Opuntia duranguensis* Britton and Rose	Whole fruit	176.86 ± 3.15 mg GAE 100 g^−1^ (FWB)	26.05 ± 0.06 mg 100 g^−^^1^	9.01 ± 0.06 mg 100 g^−^^1^	ND	290.52 ± 3.07 mg QE 100 g^−1^ (FWB)	ND	1.98 mg QE 100 g^−1^ (FWB)	[7]
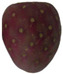	*O. oligacantha* Föster cv Borrego	Whole fruit	196.62 ± 2.94 mg GAE 100 g^−1^ (FWB)	8.67 0.13 ± 0.13 mg 100 g^−^^1^ (FWB)	3.67 ± 0.03 mg 100 g^−^^1^ (FWB)	ND	255.65 ± 2.35 mg QE 100 g^−^^1^ (FWB)	ND	4.77 ± 0.10 mg QE 100 g^−^^1^ (FWB)	[2]
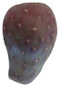	*O. oligacantha* (Förster) Ulapa	Whole fruit	278 ± 2.2	0.76 ± 0.36 mg 100 g^−^^1^	4.50 ± 0.36 mg 100 g^−^^1^	ND	ND	9.80 ± 0.22 (mmol TE 100 g^−1^)	ND	[2]

ND, no data found; (FWB), fresh weight base; (DWB), dry weight base; (mg GAE 100 g^−1^), mg equivalent of gallic acid; (mg QE 100 g^−1^), mg equivalent of quercetin; (mmol TE 100 g^−1^) Trolox (6-hydroxy-2, 5, 7, 8-tetramethychroman-2- carboxylicacid), mg VCEAC g^−1^ equivalents, as equivalent to vitamin C (mg g^−1^).
